# Characterization of a Gene Coding for the Complement System Component FB from *Loxosceles laeta* Spider Venom Glands

**DOI:** 10.1371/journal.pone.0146992

**Published:** 2016-01-15

**Authors:** Daniela Tiemi Myamoto, Giselle Pidde-Queiroz, Rute Maria Gonçalves-de-Andrade, Aurélio Pedroso, Carmen W. van den Berg, Denise V. Tambourgi

**Affiliations:** 1 Immunochemistry Laboratory, Butantan Institute, São Paulo, Brazil; 2 Institute of Molecular and Experimental Medicine, School of Medicine, Cardiff University, Cardiff, United Kingdom; Instituto Butantan, BRAZIL

## Abstract

The human complement system is composed of more than 30 proteins and many of these have conserved domains that allow tracing the phylogenetic evolution. The complement system seems to be initiated with the appearance of C3 and factor B (FB), the only components found in some protostomes and cnidarians, suggesting that the alternative pathway is the most ancient. Here, we present the characterization of an arachnid homologue of the human complement component FB from the spider *Loxosceles laeta*. This homologue, named Lox-FB, was identified from a total RNA *L*. *laeta* spider venom gland library and was amplified using RACE-PCR techniques and specific primers. Analysis of the deduced amino acid sequence and the domain structure showed significant similarity to the vertebrate and invertebrate FB/C2 family proteins. Lox-FB has a classical domain organization composed of a control complement protein domain (CCP), a von Willebrand Factor domain (vWFA), and a serine protease domain (SP). The amino acids involved in Mg^2+^ metal ion dependent adhesion site (MIDAS) found in the vWFA domain in the vertebrate C2/FB proteins are well conserved; however, the classic catalytic triad present in the serine protease domain is not conserved in Lox-FB. Similarity and phylogenetic analyses indicated that Lox-FB shares a major identity (43%) and has a close evolutionary relationship with the third isoform of FB-like protein (FB-3) from the jumping spider *Hasarius adansoni* belonging to the Family Salcitidae.

## Introduction

During evolution, two systems of immunity have arisen: innate and adaptive. The innate immune system is the oldest and found in all multicellular organisms, while the adaptive immune system, which emerged about 450 million years ago, is present only in vertebrates, except for the Agnatha [1,2]. The complement system, in mammals, plays an important role in both, innate and adaptive immune system and is composed of more than 30 serum and cell-surface components that participate in the recognition and clearance of invading pathogens. The activation of the complement system can occur by three pathways: classical, lectin and alternative that converge at the cleavage of the central complement component C3, by the C3 convertases [3].

In the alternative pathway, FB acts as the catalytic subunit of the C3 convertase; in the classical and lectin pathways, this role is played by C2. In mammals, FB and C2 share the same domain and genomic organization, with a significant amino acid similarity and, possibly, they diverged at the jawed vertebrate lineage by gene duplication [1,4]. Human FB is a modular chymotrypsin-like serine protease comprised of N-terminal region, composed of three complement control protein (CCP) domains, a linker region, a vWFA (von Willebrand factor type A) domain, and a C-terminal serine protease (SP) domain, which contains the catalytic site. The vWFA and SP domains form the fragment Bb, while the CCP1-3 and the linker form the fragment Ba. Following binding of FB to C3b, FB is cleaved by factor D into fragments Ba and Bb. FB binding to C3b depends on the CCP elements in fragment Ba and on the Mg^2+^-metal ion-dependent adhesion site (MIDAS) motif, in the vWFA domain of fragment Bb [5].

The CCP module is a domain commonly present in many mammalian complement proteins that is responsible for mediating protein-protein interactions of complement proteins or, as in factor H, to bind to self-cells. Among the three CCPs present in human FB, the third one has structural elements that are crucial for the interaction with C3b fragment.

The studies of vertebrate and invertebrate genomes revealed that many domains of mammalian complement components are found in both deuterostomes and protostomes. According to Nonaka and Kimura (2006) [2], the origin of the complement system probably occurred with the appearance of C3 and FB, the only components found in some protostomes and in cnidarians, suggesting that the alternative pathway represents the most ancient complement pathway. Whereas C3 and FB were maintained in all deuterostomes, they were lost many times, independently, in the protostome lineage, which explains the absence of these components in the insect *Drosophila melanogaster* [6] and in the worm *Caenorhabditis elegans* [7].

Since *MBL* (mannose-binding lectin), *MASPs* (MBL-associated serine proteases) and *ficolins* genes, that play a role in the lectin pathway activation, have not been identified in protostomes and echinoderms, it was suggested that these components were recruited after the emergence of chordates, about 900 million years ago. However, the recent finding of a *MASP* gene in cnidarians [8] suggests that the primitive lectin pathway could operate, besides the alternative pathway, in those animals. The agnates that are jawless vertebrates have developed only the alternative and lectin pathways of the complement system, probably due to absence of immunoglobulin genes [9]. Finally, the gene duplication events that happened in *C3/C4/C5*, *FB/C2* and *MASPs/C1r/C1s*, before the emergence of cartilaginous fishes about 600 million years ago, were important steps in the establishment of the classic pathway [2].

Genes coding for FB-like proteins have been characterized in different organisms belonging to previous lineage of chordates. FB-like sequences from the limulus *C*. *rotundicauda* (CrC2/Bf) [10] and the sea urchin *S*. *purpuratus* (SpBf) [11] have some particularities, such as the presence of five CCP domains instead of three as found in mammals, suggesting that the presence of additional CCP domains is a primitive characteristic of C2/FB proteins. In contrast, Kimura et al. (2009) [8] characterized two FB-like protein isoforms from the anemone *Nematostela vectensis* (NvBf-1 and NvBf-2): the first one contained three CCPs while the second had five CCP domains. Therefore, the Nv-Bf1 was the first FB-like gene with the same composition of CCP domains as found in vertebrates. Recently, some FB-like sequences from organisms belonging to the Arthropoda phylum were identified and multiple isoforms, containing between two and seven CCPs were found in these organisms [12]. It is possible that the composition of three CCP domains is an ancestral characteristic and that this region has undergone gene duplication or deletion in invertebrates FB. Nonetheless, it seems likely that predicted proteins presenting the same structural domains, found in anemone and limulus, are orthologues of C2 and FB genes, and probably have arisen before divergence of Cnidaria/Bilateralia [2].

When we elucidated the transcriptome of the *Loxosceles laeta* spider venom gland, in addition to finding the expression profile of the Sphingomyelinases D, the major proteins responsible for the envenomation, other EST sequences with similarity to C3 and FB-like genes, from invertebrate organisms, were identified [13]. These findings suggest that the central components of the complement system could be expressed in the venom gland of the *Loxosceles* spiders. Thus, the present work aimed to clone and characterize the FB complement component from *Loxosceles laeta* venom gland and phylogenetically analyze its deduced amino acid sequence.

## Material and Methods

### *Loxosceles* spiders and isolation of RNA

*Loxosceles laeta* spiders were collected in Campo Alegre, Santa Catarina, Brazil and kept at Immunochemistry Laboratory of Butantan Institute, São Paulo, Brazil. Eighty *L*. *laeta* female spiders were subjected to food restriction to stimulate the production of mRNA in the venom glands. After 5 days, the venom glands were collected and frozen at -80°C until use. For total RNA extraction, the Trizol reagent was used following the manufacturer’s instructions (Gibco-BRL Life Technologies, MD, USA). The authorization to access the *L*. *laeta* (permission no. 01/2009) was provided by the Brazilian Institute of Environment and Renewable Natural Resources (IBAMA), an enforcement agency of the Brazilian Ministry of the Environment.

### RT-PCR and Rapid amplification of cDNA ends (RACE)

Based on the EST sequence LLAE0889S, which we previously identified in the transcriptome of *L*. *laeta* to be similar to the complement factor B [13], specific sense and antisense primers were designed to amplify the complete gene sequence (FB sense – 5’ CGAAGCAGCTCAAGGACCAC 3’ and FB antisense – 5’ CCTTCCATCCATGCGACCAC 3’). The SMARTer RACE cDNA Amplification kit (Clontech, CA, USA), was used to amplify the *Loxosceles* FB (Lox-FB) RNA. The PCR reactions were performed using the following conditions: 2 cycles of 94°C for 30 sec, 72°C for 3 min; 5 cycles of 94°C for 30 sec, 70°C for 30 sec and 72°C for 3 min and 27 cycles of 94°C for 30 sec, 68°C for 30 sec and 72°C for 3 min.

### Cloning and sequencing of Lox-FB

Resulting products from the RACE reactions were separated in 1% agarose gel and the positive PCR products were purified using PureLink™ PCR Purification kit (Invitrogen, CA, USA) and directly cloned into pGEM-T-Easy Vector (Promega, WI, USA), at 16°C overnight, and transformed into *E*. *coli* competent Cells XL1Blue. Positively transformed cells were grown overnight at 37°C in LB (Luria Bertani) broth supplemented with 100 μg/mL ampicillin. Plasmids were isolated by Boiling Plasmid Mini Prep method [14], digested with restriction endonuclease enzyme EcoRI (New England Biolabs, MA, USA) and purified using phenol and chloroform [15].

The positive clones were sequenced from both ends with T7 (5’ TAATACGACTCACTATAGGG 3’), Sp6 (3’ TAAATCCACTGTGATATCTT 5’) and specific primers for FB (FB sense—5’ CGAAGCAGCTCAAGGGACAC 3’–and FB antisense—5’ CCTTCCATCCATGCGACCAC 3’) using Big Dye terminator Cycle Sequencing Ready Reaction Kit and automated DNA sequencer, model ABI 3100 capillary electrophoresis (Applied Biosystems, CA, USA).

### Sequence analysis and molecular modeling

All sequences were analyzed both at the nucleotide and amino acid levels using the Basic Local Alignment Search Tool (BLAST) from the National Center for Biotechnology Information (NCBI: http://www.ncbi.mlm.nih.gov/blast/BLAST.cgi). Translation and protein analyses were performed using ExPaSy tools (www.expasy.org). The deduced amino acid sequence of Lox-FB was aligned with the corresponding sequences of various animals using MEGA 6 software. On the basis of the human FB structure (PDB ID code: 2ok5.1), Swiss-model website (http://swissmodel.expasy.org/) was used to create a comparative homology model of Lox-FB [16].

### Phylogenetic analysis

All FB-like sequences used for phylogenetic analysis were downloaded from the GenBank database. Multiple sequence alignments were performed with full length open reading frame sequences using MUSCLE (Multiple Sequence Comparison by Log-Expectation) and the phylogenetic tree was constructed based on this alignment using the Maximum Likelihood (ML) algorithm available in MEGA 6 software [17]. Statistical confidence of the evolutionary analysis was assessed by bootstraps of 1000 replicates.

## Results

### Characterization of *L*. *laeta* FB

**Fig 1:**
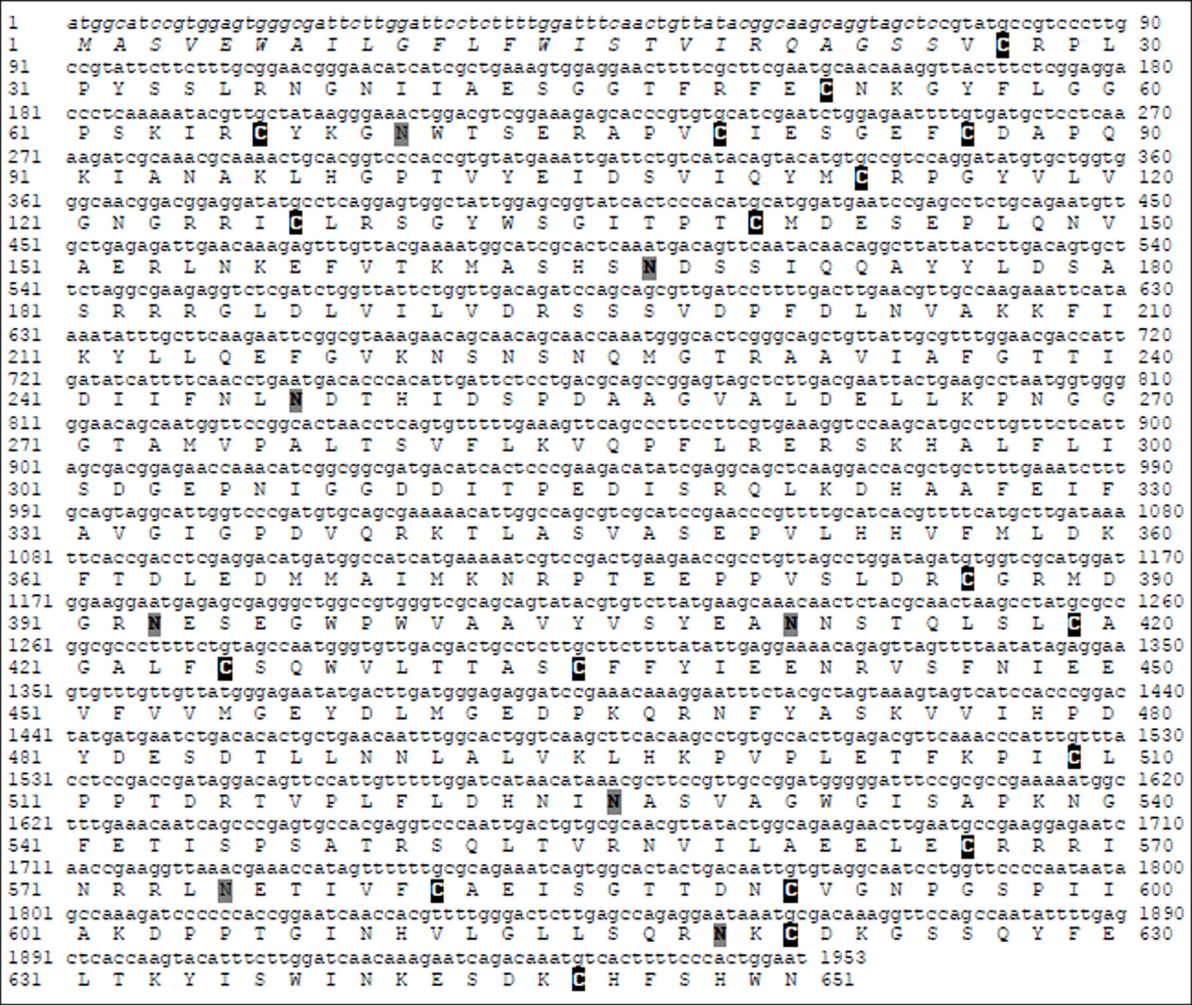
cDNA sequence and deduced amino acid sequence of Lox-FB. The ORF predicts a protein of 651 amino acids with domains of known C2 and Bf proteins. The signal peptide is in italics and comprised of the initial 25 amino acids. The cysteines and the putative N-glycosylated sites are marked in black and grey, respectively.

**Fig 2:**
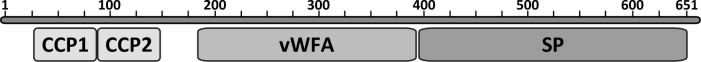
Domain structure of Lox-FB deduced protein using Basic Local Alignment Tool (BLAST).

The 5’ and 3’ RACE fragments yielded a complete reading frame (ORF) of *Loxosceles laeta* factor B-like composed of 1953 base pairs (bp) that encodes for a protein of 651 amino acids (Fig 1). The NCBI’s conserved domain database (CDD) program identified that Lox-FB has a classical domain organization, composed of two CCPs, a vWFA domain and a SP domain (Fig 2). The leader peptide signal is composed of 25 amino acids producing a mature protein of 626 amino acids. Eighteen cysteines were found, eight of them present in the CCP domains, one cysteine in the vWFA domain and nine cysteines in the SP domain (Fig 1). The deduced molecular weight of Lox-FB was predicted as 72.38 kDa and the isoeletric point as 5.73, without considering the eight putative N-glycosylation sites.

### Multiple Alignment of Lox-FB with other FB/C2-like proteins

**Fig 3:**
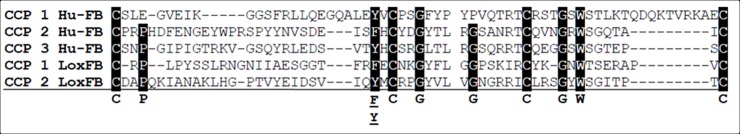
Alignment of Hu-FB and Lox-FB CCPs. The five CCPs are aligned to each other with the consensus amino acids shown in bold and at the bottom. Two disulfide bonds sustain the CCP domain and are formed between the first and third cysteine, and the second and the fourth cysteine. The alignment was done with CLUSTAL W using Bioedit v. 7.0.9.0 software.

Although the C2/FB proteins described in the literature, until now, have the same architecture of domains composition, they are different in some aspects like the quantity of CCP domains. For example, the FB-like found in some species belonging to Echinodermata phylum [11], horseshoe crab [10] and one of two isoforms present in a sea anemone [8] contains five CCP domains instead of three found in vertebrates, in which they were the first to be characterized (Table 1). Lox-FB has only two CCP domains, as FB found in bivalves [18], FB-2 centipede, FB-2 sea spider and FB-3 jumping spider [12]. Because of these differences, alignments at this position tended to be out of register in C2/FB sequences that contain more than two CCP domains. Each CCP module from Lox-FB has approximately 60 amino acids of length and there are some highly conserved residues as proline (P), glycine (G), tryptophan (W) and four cysteines (forming two disulfide-bridges; I-III and II-IV) (Fig 3).

**Table 1:** Structural features of invertebrates FB.

Species	Protein	N° of CCPs	Extra Domains	Length (aa)
*N*. *vectensis*	FB-1	3	-	708
	FB-2	5	-	858
*Ammothea sp*	FB-1	7	-	1023
	FB-2	2	-	658
*H*. *adansoni*	FB-1	7	-	1006
	FB-2	4	-	847
	FB-3	2	-	636
***Loxosceles laeta***	FB	2	-	651
*S*. *subspinipes*	FB-1	7	-	1076
	FB-2	2	-	658
*T*. *tridentatus*	C2/FB-1	5	-	889
	C2/FB-2	7	-	972
*C*. *rotundicauda*	C2/FB-1	5	-	889
	C2/FB-2	5	-	889
*R*. *decussatus*	FB	2	-	697
*S*. *purpuratus*	FB	5	-	833
*A*. *japonicus*	FB-1	5	-	913
	FB-2	5	-	865
*B*. *belcheri*	FB/C2	3	EGF-CA	752
*C*. *intestinalis*	FB-1	4	-	999
	FB-2	4	-	998
	FB-3	4	-	963
*H*. *roretzi*	FB	5	LDL_A	1084
*L*.*camtschaticum*	FB-1	3	-	763
	FB-2	3	-	749
*Homo sapiens*	FB	3	-	764
	C2	3	-	752

**Fig 4:**
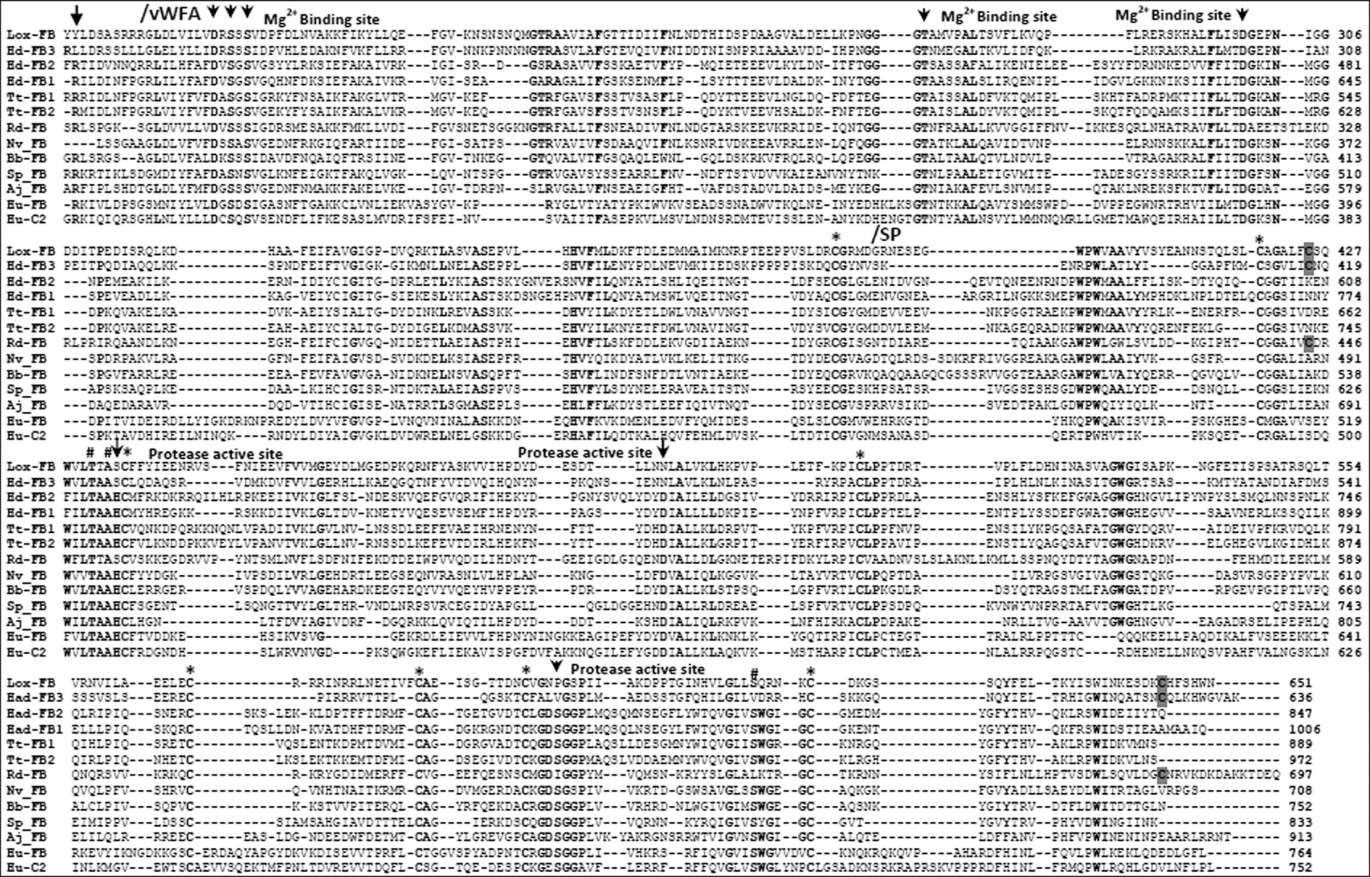
Multiple alignment of the vWFA and serine protease domains from Lox-FB with sequences of FB/C2 proteins from other organisms. The alignment was performed using MUSCLE algorithm available in MEGA software. Amino acids that are highlighted in bold indicate identical regions. The amino acids residues that are functionally important at Factor D or C1s cleavage site; the metal ion dependent binding site (MIDAS) and the protease active sites are indicated by dark arrows; the three amino acids residues (T^431^, A^433^, S^616^) that are important on stabilization of catalytic triad are indicated by sign #; the conserved cysteines residues are indicated by asterisks and the two extra cysteines present in Lox-FB, Hd-FB3 and Rd-FB are highlighted in grey. *Loxosceles laeta* (Lox-FB), *Hasarius adansoni* (Hd-FB1; HD-FB2; Hd-FB3), *Tachypleus tridentatus* (Tt-FB1;Tt-FB-2), *Ruditapes decussatus* (Rd-FB), *Nematostella vectensis* (Nv-FB), *Branchiostoma belcheri* (Bb-FB), *Strongylocentrotus purpuratus* (Sp-FB), *Apostichopus japonicus* (Aj-FB), *Homo sapiens* (Hu-C2;Hu-FB).

Analyzing the other two domains, many conserved sites were detected, such as the five amino acids involved in the binding to C3b dependent on Mg^2+^ ions present in the vWFA domain; seven cysteines and those regions close to the active site also appeared in the same position in the SP domain (Fig 4). However, none of the three amino acids residues important in the serine protease activity (catalytic triad) were conserved (Fig 4). Apparently, the classic catalytic triad of serine peptidases belonging to trypsin-like family composed of histidine (H), aspartic acid (D) and serine (S) residues [19,20] was replaced by other amino acids: serine (S), asparagine (N) and proline (P). Interestingly, Lox-FB, FB-like isoform 3 from the spider *Hasarius adansoni* and FB-like molecule of bivalve *Ruditapes decussatus* are similar, since they have only two CCP domains, two extra cysteines (highlighted in grey) and did not preserve the catalytic triad. Considering only the spider species, both of them share the same amino acids at the first and second position of the protease active site, but not the last one. Because of these differences, it could be that these proteins have lost their proteolytic activity or they have a different mechanism of activation. Despite the difference in the amino acids considered to be of importance to the enzymatic function of human FB, Lox-FB had conserved amino acids residues placed surrounding the triad, particularly Thr^54^, Ala^56^ and Ser^214^ (chymotrypsin numbering) that play an important role in stabilization of catalytic triad [20].

### 3D Structure of Lox-FB

**Fig 5:**
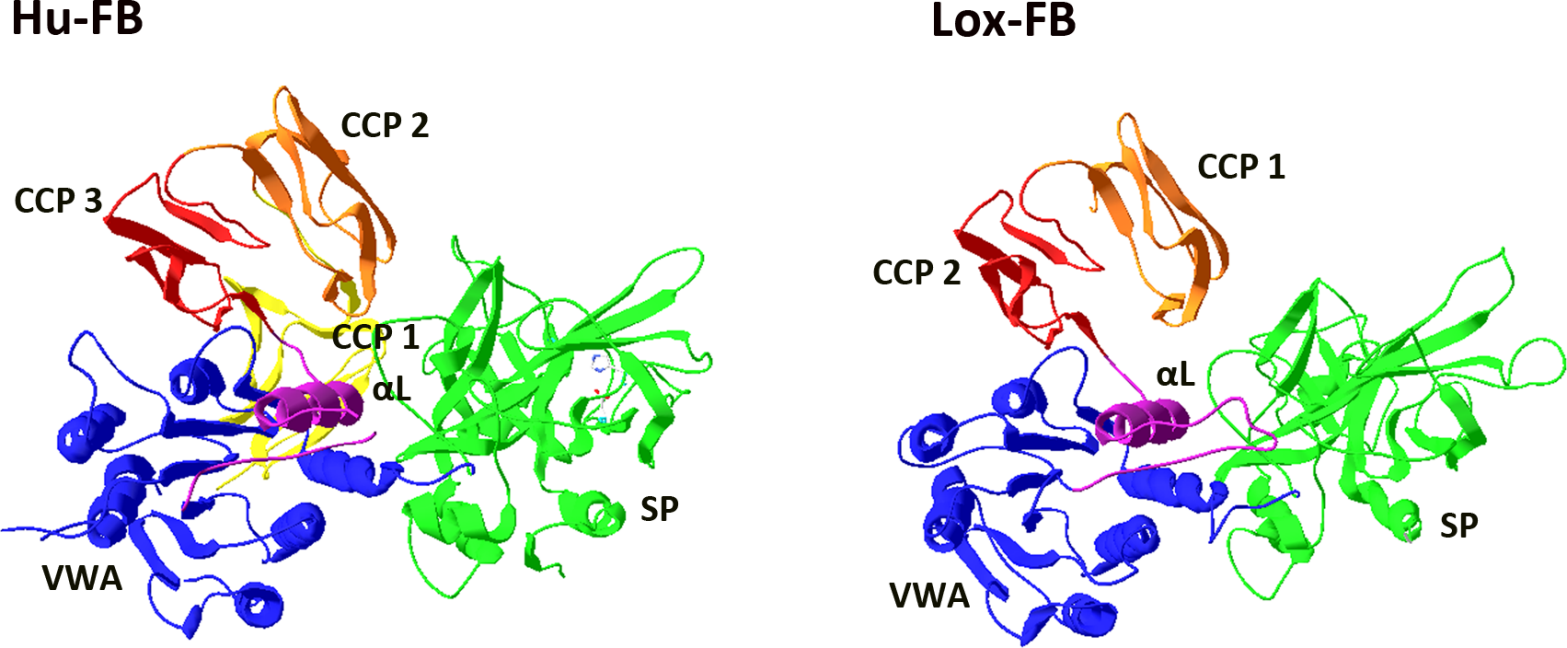
Molecular model of Lox-FB. Construction of molecular model based on structure of human factor B (PDB 2ok5.1), using the tool SWISS-Model Workspace available on http://swissmodel.expasy.org/workspace/. The further analyses were performed using the software SwissPDBViewer.

**Fig 6:**
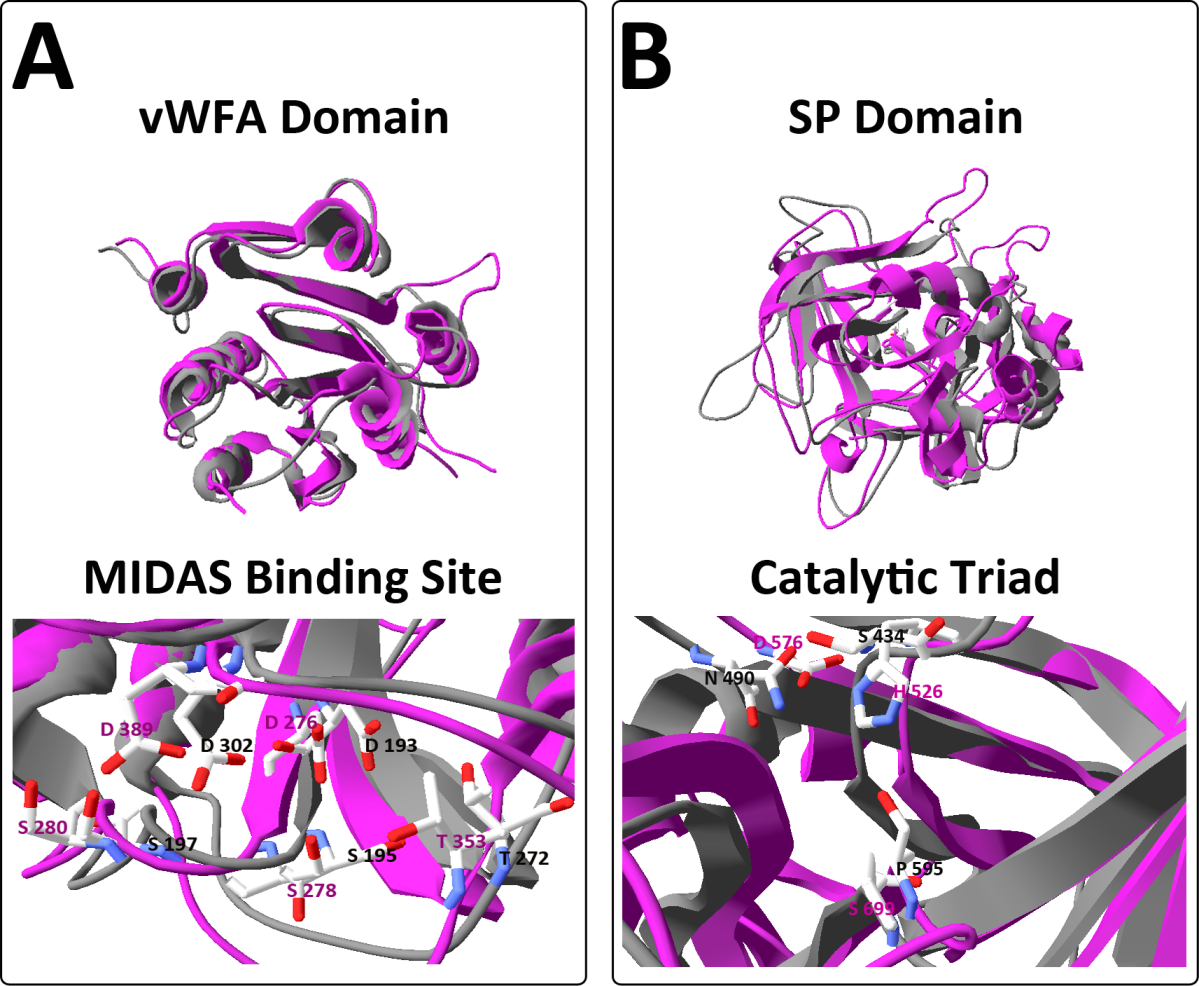
Overlap of human factor B (Hu-FB) (pink) and the *Loxosceles* factor B-like (Lox-FB) (grey). [A] vWA Domain [B] SP Domain [C] amino acids residues that comprise the catalytic triad of Hu-FB (H, D, S) and Lox-FB (S, N, P). The manipulation of models were performed using the software SwissPDBViewer.

The resulting predictive structure of Lox-FB, obtained after computational modeling using the available crystal structure of human factor B (2ok5.1), revealed 23.02% of identity and, despite differences in the number of CCP domains, a remarkable structural similarity between the CCP2-CCP3 domains of human FB and CCP1-CCP2 from Lox-FB was observed (Fig 5). Furthermore, the vWFA domain fits perfectly with human FB, since the six major α-helices surrounding a central twisted β-sheet are present and conserved at the same positions with minor differences on the conformation of the loop folds. As well as linear alignment, the amino acids that represent the metal ion-dependent binding site (MIDAS) are conserved and occupy the same positions on the 3D structure (Fig 6A). With respect to the serine protease domain (SP), the overlap was not as perfect as that observed for the vWFA domain; nevertheless, the regions that constitute the secondary structures of the β sheet are overlapping (Fig 6B). The three amino acids that comprise the catalytic triad of human factor B (H, D, S) are aligned with the same three amino acids (S, N, P) from Lox-FB observed in the alignment of the primary sequences (Fig 6B).

### Phylogenetic analysis

**Fig 7:**
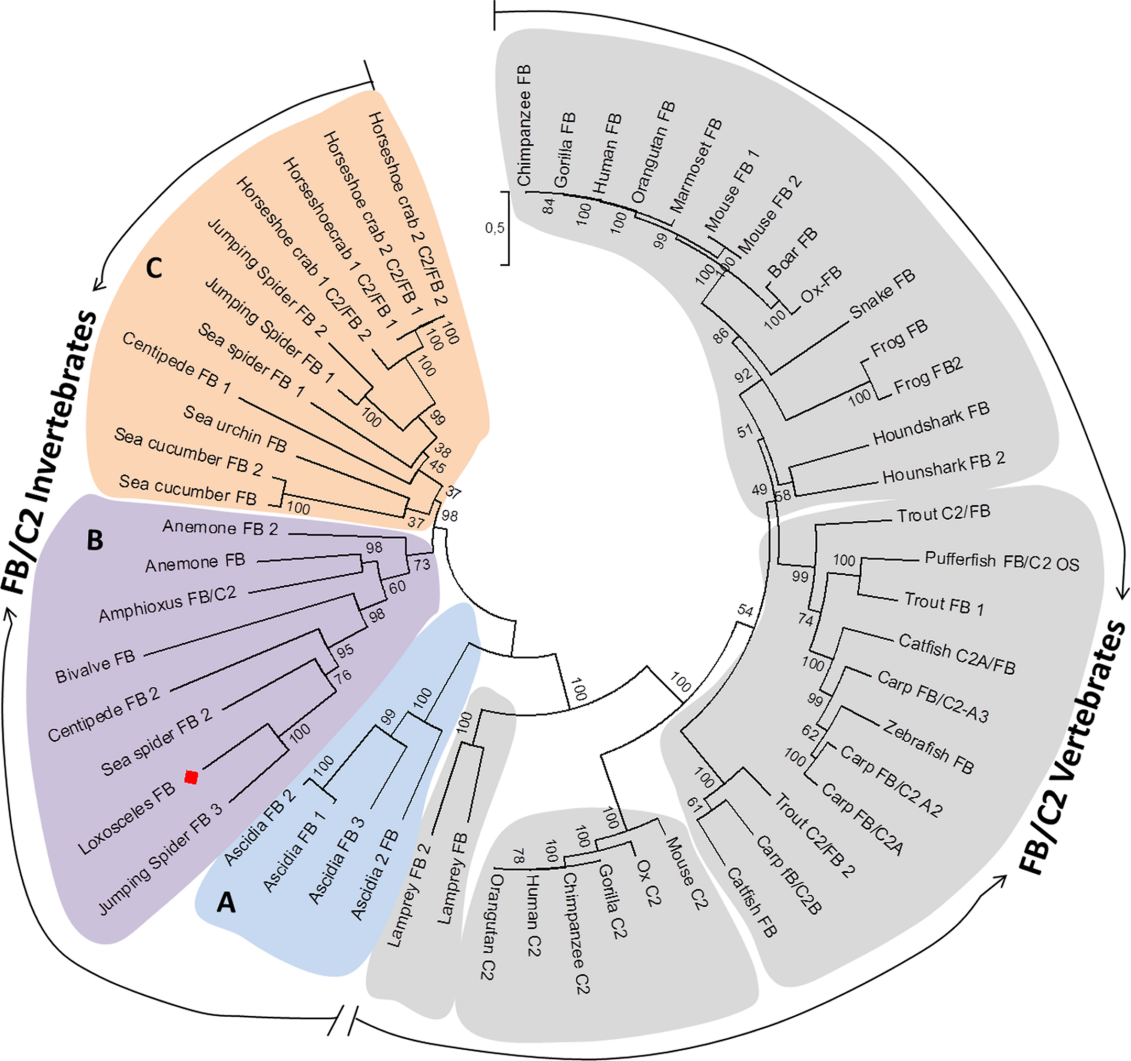
Phylogenetic relationships among Lox-FB and reported C2 and FB proteins. The tree was constructed based on alignment done with MUSCLE and Maximum Likelihood (ML) algorithm using MEGA 6 software. Statistical confidence of the evolutionary analysis was assembled by bootstraps of 1000 replicates. Sequences were obtained from GenBank Database. Gorilla FB (Q864V9), Chimpanzee FB (Q864W0), Human FB (P00751), Orangutan FB (Q864W1), Marmoset FB (JAB17376.1), Mouse FB-1 (P04186), Mouse FB-2 (B8JJM5), Ox-FB (P81187), Boar FB (ABX82825.1), Snake FB (AAR21601.1), Frog FB (BAA06179.1), Frog FB-2 (AAI70192.1), Trout C2/FB (ADY68777.1), Trout FB 1 (AAC83699.1), Pufferfish FB/C2 OS (CAD21938.1), Catfish FB/C2 A (AEW10545.1), zebrafish FB (AAB19093.1), Carp FB/C2 A3 (BAB32650.1), Carp FB/C2 A2 (BAA78416..1), Carp FB/C2 A (BAA34706.1), Houndshark FB (BAB63203.1), Houndshark FB 2 (BAF62177.1), Catfish FB (AEW38662.1), Carp FB/C2 B (BAA34707.1), Trout C2/FB 2 (BAB19788.1), Mouse C2 (P21180), Ox C2 (Q3SYW2), Orangutan C2 (Q8SQ74), Gorilla C2 (Q863A0), Human C2 (P06681), Chimpanzee C2 (Q8SQ74), Lamprey FB (BAA02763.1), Lamprey FB 2 (BAG66069.1), Ascidia FB (AAK00631.1), Ciona FB-3 (BAD89301.1), Ciona FB-1 (BAD89299.1), Ciona FB-2 (BAD89300.1), Amphioxus FB/C2 (ABY28382.1), Anemone FB (BAH22726.1), Anemone FB 2 (BAH22728.1), Bivalve FB (ACQ91095.1), Sea cucumber FB 2 (AEP68015.1), Sea cucumber FB (ADX36428.1), Sea urchin FB (AAC79682.1), Horseshoe crab 1 C2/FB 1 (BAM15262.1), Horseshoe crab 1 C2/FB 2 (BAM15263.1), Horseshoe crab 2 C2/FB 1 (AAV65032), Horseshoe crab 2 C2/FB 2 (ABK30937.1), Jumping spider FB1 (BAR45590-1),Jumping spider FB2 (BAR45591.1), Jumping spider FB3 (BAR45592.1), sea spider FB 1 (BAR45606.1), sea spider FB 2 (BAR45607.1), centipede FB 1 (BAR45616.1), centipede FB 2 (BAR45617.1).

Lox-FB showed similarity with complement proteins sharing 25% and 26% identity with human FB and C2, respectively (Table 2). Values obtained with invertebrates FB/C2 proteins exhibited a similarity ranging from 22% to 43% of identity. To investigate the evolutionary history of FB and C2 complement proteins and to determine how *Loxosceles* spiders fit into this picture, 56 FB and C2 sequences were used and subjected to phylogenetic analysis. An unrooted phylogenetic tree was constructed and resulted in two differentiated groups, vertebrate and invertebrate proteins (Fig 7). Considering the vertebrate proteins, there is a clear separation between C2 and FB proteins, except for some fishes FB/C2 sequences. The lamprey FB (jawless vertebrate) is positioned outside of the jawed vertebrate FB and C2 components suggesting that it represents the ancestral group of higher vertebrates FB and C2 proteins. Considering the branch represented by invertebrates, there are three main groups: the first one is represented by ascidians FB-like sequences (group A) which are possibly the closest ancestor of FB/C2 vertebrate sequences. The second was called group B, which comprises Lox-FB and FB-like sequences from cnidarians, amphioxus and bivalve; Lox-FB is located at the same branch of FB proteins (isoform 2) as the centipede and sea spider, and at the same sub-branch of the jumping spider (isoform 3) (Bootstrap 100%). Finally, group C, consists of FB-like sequences from echinoderms and eight isoforms found in five species belonging to the Arthropoda phylum.

**Table 2:** Pairwise comparisons of Lox-FB vs others FB and C2 proteins.

Organism gene	N° de Access	I (%)*	S (%)**	*Score*	*E value*
FB-3 *H*. *adansoni*	dbj||BAR45592.1	43	62	483	1e-158
FB-2 *Ammothea sp*	dbj|BAR45607.1	35	52	349	1e-106
FB/C2 *B*. *belcheri*	gb|ABY28382.1	30	48	264	3e-74
FB precursor *N*. *vectensis*	dbj|BAH22727.1	30	46	237	1e-64
FB-2 *S*. *subspinipes*	dbj|BAR45617.1	28	44	231	9e-63
FB precursor *N*. *vectensis*	dbj|BAH22728.1	28	46	224	2e-59
FB-*like R*. *decussatus*	gb|ACQ91095.1	27	47	206	7e-54
FB-1 *Ammothea sp*	dbj|BAR45606.1	26	41	185	8e-46
FB-2 *A*. *japonicus*	gb|AEP68015.1	27	42	180	1e-44
FB *A*. *japonicus*	gb|ADX36428.1	27	44	180	1e-44
FB-1 *H*. *adansoni*	dbj|BAR45590.1	25	45	171	2e-41
FB *S*. *purpuratus*	gb|AAC79682.1	28	42	169	5e-41
FB *L*. *japonicum*	dbj|BAA02763.1	23	40	154	3e-36
C2/FB-2 *T*. *tridentatus*	gb|BAM15263.1	24	42	149	3e-34
FB-1 *S*. *subspinipes*	dbj|BAR45616.1	24	42	142	1e-31
*FB Danio rerio*	gb|AAH97100.1	25	43	140	2e-31
C2/FB-1 *T*. *tridentatus*	gb|BAM15262.1	22	41	139	5e-31
FB/C2B *G*. *cirratum*	gb|AAY56127.1	25	41	135	5e-30
C2/FB-2 *C*. *rotundicauda*	gb|ABK30937.1	23	42	131	1e-28
C2/FB-1 *C*. *rotundicauda*	gb|AAV65032.2	23	42	131	2e-28
FB/C2A *C*. *carpio*	gb|BAA34706.1	25	41	126	5e-27
FB/C2A-3 *C*. *carpio*	gb|BAB32650.1	25	44	125	8e-27
FB-*2 precursor M*. *musculus*	gb|NP_001136178.1	24	42	125	8e-27
FB-1 *precursor M*. *musculus*	gb|NP_032224.2	24	42	125	1e-26
C2 *H*. *sapiens*	gb|AAB67975.1	26	41	119	4e-32
FB *H*. *sapiens*	gb|CAA51389.1	25	41	121	8e-33

*Identity (I) was calculated based on percentage of identical amino acids at per column/position in the alignments.

**Similarity (S) was calculated as the percentage of identical plus similar residues, which are conservative substitutions.

## Discussion

The studies of the evolution of the complement system have progressed in the last years and due to technological advances, including the genomic and transcriptomic methodologies, resulting in the increased possibility of discovering genes related to the complement system in invertebrates. At the present moment, there is more information about the existence of *C3* genes than of *FB* genes, however, it is known that the *FB* gene is missing from genome sequences of cnidarians hydra, the nematode *C*. *elegans* and several species of insects. These findings suggest that the origin of the *FB* genes probably has occurred before the divergence between Cnidaria and Bilateralia, more than one billion years ago, and the absence of these complement genes in cnidarian and in some protostome lineages can be explained due to a secondary loss [21,2]. The present work confirms this hypothesis, since a *FB-*like gene was also found expressed in *Loxosceles* spider venom gland. This finding represents the first identification of a factor B homologue from a *Loxosceles* spider (Arachnida, Sicaridae) and similar to the vertebrate FB/C2 proteins, Lox-FB is a mosaic protein composed of CCP, vWFA and serine protease domains.

Many studies have indicated the existence of a less complex complement system, named the “archeo-complement system” involving C3-like proteins associated to factor B-like proteins as it was described in lamprey [22], sea urchin [11], horseshoe crab [10], ascidians [23], amphioxus [24], sea anemones [8], bivalve [18] and sea cucumber [25]. Factor B is a component of the alternative pathway and since this gene was found in organisms belong to primitive lineage (cnidarians and protostomes), it is possible that these organisms are endowed with alternative pathway activity.

All invertebrate C2/FB proteins, characterized so far, preserve the same classic architecture domain found in related vertebrate proteins. Despite of some particularities in N-terminal region such as extra CCP domains, observed in sea urchin and horseshoe crab, or the additional domains, as low density lipoprotein receptor like domain (LDL_A), found in ascidians or epidermal growth factor-like domain (EGF_CA) in amphioxus, most of all these C2/FB proteins have conserved the regions that play important role for activation of this protein and because of that they are considered orthologues of the mammalian FB and C2.

Highly conserved regions were identified in Lox-FB such as CCP consensus sequence, Mg^2+^ binding sites in the vWFA domain and conserved positions near to the catalytic center within the SP domain. However, considering the domain architecture, Lox-FB has only two CCP domains, one less than observed in vertebrates, but is similar to FB-3 present in the jumping spider *Hasarius adansoni*, the centipede *Scolopendra subspinipes japonica* FB-2, the sea spider *Ammothea sp* FB-2 and the bivalve *Ruditapes decussatus* FB [12,18]. The CCP module is a domain commonly present in many mammalian complement proteins and is responsible for the interaction of complement proteins, with each other and their respective regulators and receptors, but also for the binding of e.g. factor H to human cells. Pathogens often mimic or capture CCP-like molecules to avoid detection and destruction by the complement system and can also use CCP-containing molecules to gain entry into the cell (*e*.*g* EBV binding to CR2) [26]. Not much of the roles of CCPs in pathogen evasion/protection in invertebrates is known, but recently, some studies demonstrated a role for CCPs in preventing lethal flaviviral infection of mosquitoes that are responsible for transmission of e.g. Dengue fever and Yellow fever. The mosquitoes are not affected by these viruses themselves, while they can transmit the disease to humans. The mosquito *Aedes Aegypti* was found to contain a neural factor AaHig that contained 5 CCP domains and that functions as viral recognition factor interacting with surface proteins of dengue virus (DENV) or Japanese encephalitis virus (JEV), thereby preventing the flaviviral entry into the mosquitos neural cells [27]. Another study showed that a scavenger receptor binds to DENV via CCP modules and indirectly helps to control flavivirus infection by inducing antimicrobial peptides [28]. Thus, even if the main role of CCP domains in invertebrates FB is to interact with C3b fragment and cause pathogen opsonization, the possibility cannot be excluded that they may also function as a pathogen recognition factors in the spider. However, a major difference between the mosquitos and the spiders is that the mosquitos are vectors in the transmission of viruses and other pathogens, and thereby it is essential that they are protected against the pathogen themselves. Such a role for spiders has not been described yet and thus no evolutionary pressure may have been present to evolve such a role for the CCP-containing spider molecules.

Factor B belongs to family S1 of clan PA of peptidases, since it has a serine protease domain that bears the chymotrypsin fold and almost all representatives of this class utilize the canonical catalytic triad represented by Asp^102^, His^57^ and Ser^195^ (chymotrypsin numbering) [20]. Despite of many similarities in the secondary structures between human FB and Lox-FB, the classical catalytic triad was not found, however, the adjoining amino acids were conserved, but whether Lox-FB has proteolytic activity remains to be investigated. It is possible that the amino acids present at the putative catalytic site may form an active site or this protein corresponds to a Lox-FB inactive isoform. We only found one FB-like molecule in the *Loxosceles* venom gland, but we cannot exclude that the *Loxosceles* spiders have other FB isoforms that possess a conserved triad catalytic in their hemolymph.

Thus far, in invertebrates, there is no clear information about specific proteolytic activity of an alternative pathway (AP) convertase C3bBb-like and how it is assembled. A serine protease activity in horseshoe crab plasma is triggered by PAMP molecules, such as LTA and LPS in Mg^2+^ and Ca^2+^-dependent manner, however, it is not clear if CrC2/Bf participates directly in CrC3 activation or if it has to be activated by other serine protease similar to FD vertebrate complement [10]. Le Saux and collaborators demonstrated a new role played by CrC2/Bf that is able to binding to the three members of PRRs: galactose-binding protein (GBP), Carcinolectin-5 (CL5) and C-reactive protein (CRP), promoting their assembly on pathogens and, consequently, activating the complement system [29]. These findings also suggest that CrC2/Bf could function as a MASP counterpart, participating in putative lectin pathway.

Although the horseshoe crab complement system is being studied with great depth considering proteolytic activities, most data derived from invertebrates FB/C2-like is represented by characteristics based on their putative structures. In other words, there is no experimental evidence that those isoforms that did not retain the classic catalytic triad actually had lost their proteolytic activity.

Furthermore, it is possible that in the *Loxosceles* complement system there is another mechanism of activation, independently of factor B, as observed in the horseshoe crab *Tachypleus tridentatus*. It was demonstrated that these organisms are endowed by a serine protease named Factor C, originally characterized as an LPS-sensitive initiator of hemolymph coagulation stored within the hemocytes, and that this factor could act as a C3 convertase on the surface of invading Gram-negative bacteria in the initial phase of complement activation [30]. Perhaps, there is also a component similar to the factor C in the hemolymph or the venom gland from *Loxosceles*, however, the activity of Lox-FB should be evaluated to understand if it has physiological roles in the *Loxosceles* complement system activation. Recently, Tagawa et al. (2012) [31] characterized two isoforms of factor B from *Tachypleus tridentatus* (TtC2/Bf-1 e TtC2-Bf2) and both of them were indispensable for TtC3b deposition on Gram-positive bacteria and fungi. Even though, they have not characterized a factor D-like serine protease in horseshoe crab, and because of this they suggested that other components, such as plasma lectins, which could be important for recruitment of the C3bBb-like on the surfaces of Gram positive bacteria and fungi. Then, it seems that the mechanism of activation of horseshoe crab complement system is different when compared to mammals and maybe the *Loxosceles* spider has the same pattern of activation.

According to phylogenetic analysis, there is a divergence between the proteins present in vertebrates and in invertebrates. At the branch represented by components factor B and C2 from vertebrates, the lamprey FB appears as sister group of vertebrates, indicating that the gene duplication events happened before the origin of jawed vertebrates. This configuration of phylogenetic tree is in agreement with the absence of classical pathway in jawless vertebrates, since they do not have immunoglobulins genes [9]. Almost all sequences from invertebrates have more than one isoform and some of them are grouped at the same clade (group C) as observed for the two isoforms (1 and 2) of the limulus *Tachypleus tridentatus* (horseshoe crab 2 C2/FB), sea cucumber *Apostichopus japonicus* and jumping spider *Hasarius adansoni*. However, in some species that expressed different FB-like isoforms, their isoforms did not locate to the same group; for instance, some isoforms of FB-like sequences from the centipede, sea spider and the jumping spider were grouped together in group B in which Lox-FB is also found, while other isoforms of the same species were grouped in group C.

The phylogenetic history of a protein does not always follow the evolution of the species because of different selective pressures. Along with the type of pathogen that the organism is infected with, other factors as adoption of different habitats, life histories and complexity will influence immune system design and mode of action and evolution [32]. Considering the whole organism, there are many types of selective pressures that influence the survival as, for instance, climate changes, predation, food availability and infections. Factor B is a protein related to the immune response and the selective pressures worked on it are mainly represented by recurrent infections, to which the organisms were exposed. Therefore, it is possible that the shell clam *Ruditapes decussatus*, amphioxus *B*. *belcheri* and the spider *Loxosceles laeta* have been infected by similar pathogens that express the same molecular patterns, which explains the distribution of these species at the same group on phylogenetic tree. Further studies will be necessary to understand the nature of the protein Lox-FB and to investigate how it interacts with other complement proteins possibly present in *Loxosceles* hemolymph. Knowing these aspects could contribute to better understand the defense mechanisms of *Loxosceles* spiders in the context of immunologic responses.
